# Distributive Shock in Erythrodermic Psoriasis Treated With Norepinephrine and Vasopressin: A Case Report

**DOI:** 10.7759/cureus.37728

**Published:** 2023-04-17

**Authors:** Mehrdad Karimi, Anas Zaher, Moshe Bressler, Sabrina Saleem

**Affiliations:** 1 Internal Medicine, NewYork-Presbyterian Brooklyn Methodist Hospital, Brooklyn, USA

**Keywords:** intensive care unit (icu), norepinephrine (ne), cerebrospinal fluid (csf), magnetic resonance imaging (mri), computed tomography (ct), human immunodeficiency virus (hiv), interleukin (il), total body surface area (tbsa), erythrodermic psoriasis (ep)

## Abstract

Distributive shock and hypothermia are two unusual and potentially fatal complications of erythroderma, a rare complication of psoriasis. Very few cases of erythrodermic psoriasis have been reported, particularly in the United States, which may pose a diagnostic challenge for internists. We present a case report of distributive hemodynamic instability and hypothermia in a 61-year-old female who initially presented with acute altered mental status thought to be related to an infectious etiology.

## Introduction

Psoriasis is a common chronic inflammatory skin condition involving the epidermis layer, which affects about 3% of Americans [1]. Erythrodermic psoriasis (EP) is a rare, severe, and potentially fatal variant involving 75%-90% of the total body surface area (TBSA). EP occurs in less than 3% of patients with psoriasis, and the average age of onset is about 48 years [2,3]. As in different psoriasis variants, EP is a complex, heterogeneous disease with multiple environmental, genetic, and autoimmune aspects. Interleukins IL-12, IL-17, and IL-23 are the major signaling pathways affected in EP, and recent biologics have been developed to target these pathways [4].

We present a rare complication of EP in a 61-year-old African American female who presented with altered mentation, hemodynamic instability, severe hypothermia, and an exfoliative erythematous rash that was diagnosed with new-onset psoriasis.

## Case presentation

**Figure 1:**
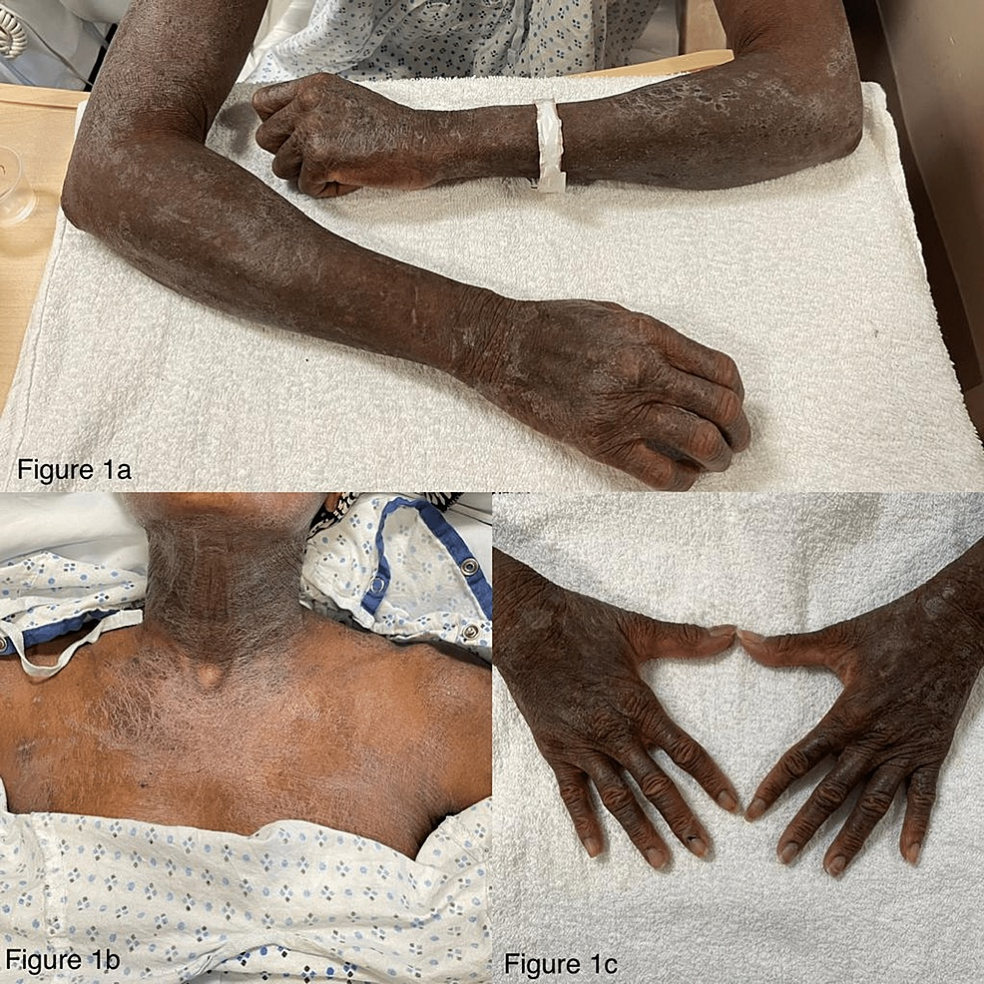
Diffuse EP Diffuse EP in a 61-year-old female with type V skin, diffuse scaly pruritic plaques on bilateral upper extremities, notable on the extensor surfaces (a). Hyperpigmented lichenified pruritic plaques on the anterior neck and chest (b). Hyperpigmented plaques on bilateral dorsal hands with nail pitting seen throughout all nailbeds (c). EP, erythrodermic psoriasis

A 61-year-old domiciled female with a past medical history of glaucoma was admitted for altered mentation (alert and oriented times zero) and a diffuse, scaly skin rash with scattered pustules covering 90% of her total body surface area (Figure 1). Within 24 hours, she developed hypothermia (28°C), hypotension (80s/50s), and sinus bradycardia (40s-50s). On day three of hospitalization, she was upgraded to the intensive care unit for vasopressor support and warming blankets. 

An extensive workup was collected, including a septic workup (lactate, procalcitonin, chest x-ray, blood cultures, and urine cultures), thyroid function tests, lumbar puncture, an autoimmune panel, a connective tissue panel, and a heavy metal panel. These tests were all grossly unremarkable. Tests for human immunodeficiency virus (HIV), syphilis, leptospirosis, and atypical infections were also negative. The patient was started on empiric antibiotics (vancomycin and piperacillin-tazobactam) and stress dose hydrocortisone (100 mg every 8 hours). Computed tomography (CT) and magnetic resonance imaging (MRI) of the brain with and without contrast were negative for any acute or chronic pathologies. Echocardiogram was performed, and the results were unremarkable. The patient had a diffuse, scaly, hyperpigmented excoriated rash, involving more than 90% of TBSA. On the fifth day of hospitalization, a skin punch biopsy of the shin was performed to rule out mycosis fungoides.

**Figure 2:**
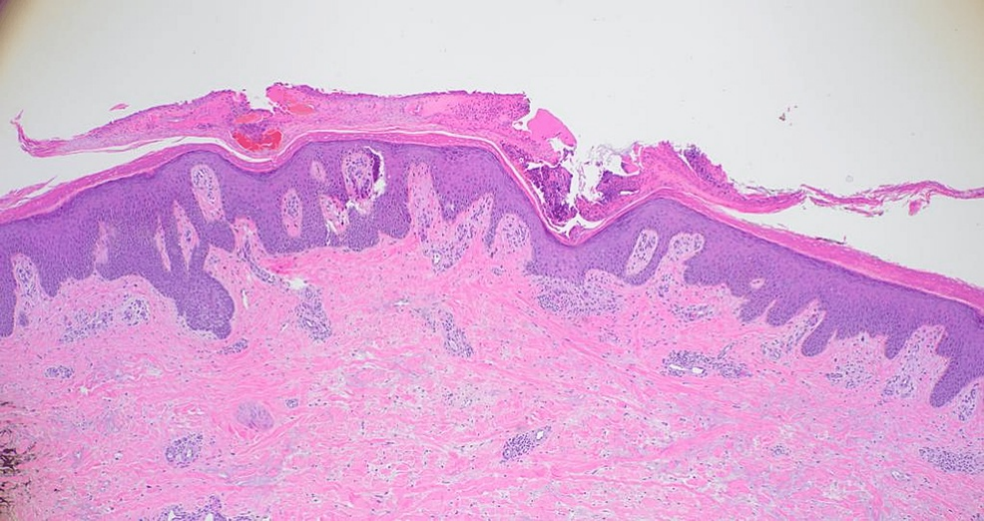
Histology 4x Zoom Histology showing psoriasiform dermatitis with acanthosis, parakeratosis, elongation of the rete ridges, perivascular infiltrates, Munro microabscesses within a thickened corneal layer, and superficial crust formation.

Skin pathology results were consistent with psoriasiform dermatitis with hyperkeratosis and crust formation consistent with psoriasis (Figure 2). The patient’s hemodynamics and mental status improved after a course of vancomycin and piperacillin-tazobactam. She was able to be weaned off vasopressors and transferred to the general medical floor. She continued to be intermittently hypothermic for a few days; however, her temperature eventually normalized. Adrenal insufficiency was ruled out due to normal AM cortisol and cosyntropin-stimulating tests. Overall, the patient responded well to vasopressors, which were discontinued after less than 24 hours. Her mental status improved back to baseline by day five of the hospitalization. She was started on triamcinolone cream 0.1%, and her skin lesions improved significantly. The patient’s exfoliative rash eventually improved after a few weeks, and she was discharged on triamcinolone cream, and the hydrocortisone was slowly tapered as per rheumatology recommendations.

## Discussion

Very few cases of EP have been reported in the United States, which can lead to diagnostic challenges, especially in rural areas where access to dermatology is limited. EP is a rare, incurable, and potentially fatal variant of psoriasis if left untreated [5]. Complications arising from EP can result in hemodynamic, metabolic, and thermoregulatory instability. Defects in the skin barrier pose additional risks for infection and, therefore, can lead to sepsis [6]. Fluid resuscitation is crucial in the initial steps of managing distributive shock. Due to disproportionate vasodilation, it is also vital to increase cardiac output to augment tissue perfusion. Vasopressors may be required for fluid-unresponsive patients due to their alpha- and beta-adrenergic receptor properties. Vasopressors augment cardiac output by increasing systemic vascular resistance and inotropy. Our patient was started on norepinephrine (NE) for hypotension and then vasopressin was added due to the increased NE requirement above 10 mcg/min. NE was the vasopressor of choice due to its effects on the alpha- and beta-adrenergic receptors. It acts by vasoconstriction, which increases the resistance to blood flow, thereby increasing blood pressure. Systemic steroid use is highly controversial for the treatment of psoriasis, as exacerbation of erythrodermic status after withdrawal has been known to occur [7]. However, some reports have shown that steroid use can be helpful in acute cases or exacerbations. However, due to the rarity of this condition, there are no documented treatment guidelines for EP. 

## Conclusions

We presented a patient who was admitted for worsening mental status and was soon found to be in distributive shock and severe hypothermia due to vasodilation. She was treated promptly and was back to her baseline mental status prior to discharge. It is important to work with a dermatologist (when possible) or other healthcare providers to manage EP and prevent recurrences. Early treatment and ongoing management can help to prevent serious complications and improve the quality of life for individuals with this condition. The treatment approach will depend on the severity of the condition, the overall health of the patient, and other individual factors.
